# Ferritin as an Inflammatory Marker in Pediatric Metabolic Syndrome: Links to Obesity and Liver Ultrasound Alterations

**DOI:** 10.3390/ijms26083793

**Published:** 2025-04-17

**Authors:** Mihaela-Andreea Podeanu, Ștefănița Bianca Vintilescu, Raluca Elena Sandu, Claudiu Marinel Ionele, Carmen Elena Niculescu, Mirela-Marinela Florescu, Elena-Loredana Șelaru, Mioara Desdemona Stepan

**Affiliations:** 1 Doctoral School, University of Medicine and Pharmacy of Craiova, 200349 Craiova, Romania; podeanu.andreea11@gmail.com; 2 Department of Infant Care, Pediatrics and Neonatology, University of Medicine and Pharmacy of Craiova, 200349 Craiova, Romania; vintilescubianca92@gmail.com (Ș.B.V.); carmen.niculescu@umfcv.ro (C.E.N.); loredana.selaru@gmail.com (E.-L.Ș.); desdemona.stepan@umfcv.ro (M.D.S.); 3 Department of Biochemistry, University of Medicine and Pharmacy of Craiova, 200349 Craiova, Romania; 4 Department of Gastroenterology, University of Medicine and Pharmacy of Craiova, 200349 Craiova, Romania; claudiu.ionele@umfcv.ro; 5 Department of Pathology, University of Medicine and Pharmacy of Craiova, 200349 Craiova, Romania; mirela.florescu@umfcv.ro

**Keywords:** metabolic syndrome, serum ferritin, hepatic steatosis, low-grade inflammation, serum iron

## Abstract

This study analyzed the relationship between obesity, metabolic syndrome (MetS) and its individual components, iron metabolism, and hepatic alterations in a pediatric group of patients. We mostly concentrated on the role of serum ferritin as a marker of inflammation. We conducted a retrospective study, in which we determined the presence of MetS and hepatic ultrasound changes in a cohort of 68 pediatric patients and examined the changes in serum iron and ferritin levels. MetS prevalence was significantly higher in obese children (64%) compared to those with average weight (11.1%). Abdominal circumference, triglycerides, and high-density lipoprotein cholesterol were the most relevant MetS criteria. Serum iron levels were significantly lower, while ferritin levels increased proportionally with MetS number of components. Liver ultrasound findings confirmed a strong association between hepatic steatosis and MetS, with advanced ultrasonographic scores correlating with increased ferritin and serum iron deficiency. These results reinforce the interplay between iron metabolism and inflammation in pediatric MetS. Given this study’s unicentric design and limited ethnic diversity, further large-scale, longitudinal studies are needed to confirm these findings and improve early screening strategies for pediatric metabolic complications.

## 1. Introduction

Obesity is defined as the excessive accumulation of fat tissue with detrimental effects on health [1]. The prevalence of obesity continues to rise globally, including among children and adolescents. Between 1990 and 2022, the global percentage of children and adolescents aged 5–19 living with obesity increased from 2% to 8% [2]. It is a complex, multifactorial disease requiring multi-strategy lifelong treatment, being a burden on the medical system all over the world [1]. This rising trend in childhood obesity is closely related to an increased risk of developing metabolic syndrome (MetS), type 2 diabetes, and cardiovascular disease later in life [3]. Children who are overweight are more likely to remain with excess weight as adults, further contributing to their risk of developing serious health complications. Early intervention is crucial in addressing childhood obesity and mitigating its long-term impact on their health and quality of life [4].

MetS and obesity among children and adolescents from Europe and United States have demonstrated significant concomitant increases in prevalence across countries and age groups [5,6]. Childhood obesity is strongly linked to reduced insulin sensitivity, a key feature of MetS [7]. Chronic inflammation may represent a triggering factor in the origin of MetS. Overnutrition, physical inactivity, and ageing can result in cytokine hypersecretion and can eventually lead to insulin resistance and diabetes in genetically or metabolically predisposed individuals [8].

The importance of chronic low-grade inflammation in the pathology of numerous age-related chronic conditions is now clear. An unresolved inflammatory response is likely to be involved from the early stages of disease development [9]. Chronic low-grade inflammation, closely linked to obesity, plays a pivotal role in the development of MetS in children, primarily by triggering mechanisms leading to insulin resistance and increased cardiovascular risk. Evidence suggests that children may be more vulnerable to oxidative stress than adults, amplifying the long-term impact of inflammation on metabolic health. Similar to adults, obese children exhibit altered levels of cytokines and adipokines, reflecting shared inflammatory mechanisms across age groups. Importantly, diet-induced weight loss has demonstrated anti-inflammatory effects, leading to significant improvements in metabolic parameters, cytokine levels, and lipid profiles. This underscores the critical role of addressing inflammation and its mediators in reducing the progression of MetS during childhood [10,11].

Iron is also significantly involved in the pathogenesis of insulin resistance [12]. Ferritin plays a critical role in iron metabolism and cellular health by regulating iron storage and preventing iron-mediated oxidative damage [13], as it sequesters excess intracellular iron, storing it in a redox-inactive form [14]. Moreover, ferritin serves as an acute phase reactant, which means its levels are associated with inflammatory, immunological, chronic, and malignant conditions [15,16,17,18].

Although elevated serum ferritin levels are linked to insulin resistance and are associated with MetS in both adult and pediatric populations [12,13,19,20], their clinical utility as a diagnostic or prognostic marker remains unproven.

The hepatic manifestation of MetS is believed to be nonalcoholic fatty liver disease (NAFLD), which recent data identify as one of the most prevalent chronic liver diseases in children and adolescents [21]. The shift from the term NAFLD to MAFLD (metabolic-associated fatty liver disease) was proposed in 2020, as it reflects the systemic nature and interplay between these conditions, with metabolic abnormalities like insulin resistance, adipokine secretion, and inflammation driving both hepatic and extra-hepatic complications [22]. Screening in patients with metabolic risk factors involves imaging techniques like liver ultrasound, alongside fibrosis scores and biological assessments like liver function tests, to confirm steatosis and identify those at risk of advanced disease or requiring further evaluation [23]. Ferritin can be a potential indicator of disease severity in NAFLD [24].

Further research is needed to confirm ferritin’s potential as a marker for identifying children at risk of MetS, as elevated levels may reflect both iron storage and the inflammation and oxidative stress driving fatty liver disease progression [24,25].

The aim of the present study is to explore the relationship between serum ferritin, serum iron, MetS, obesity, and liver ultrasound changes in a cohort of children and adolescents. Moreover, we want to explore the importance of ferritin as a marker in detecting inflammation in pediatric MetS.

## 2. Results

### 2.1. Obesity Group vs. Average Weight

The clinicoepidemiological analysis of the study group included 68 patients; 50 cases (73.5%) were classified as obese, and 18 cases (26.5%) showed average weight for age and gender. The median age of the studied group was 10.5 years (8–12), with a body mass index (BMI) of 25.65 kg/m^2^ (19.1–29.3). The majority were males from urban areas, numbering 50 cases (73.5%) and 37 cases (54.4%), respectively.

The classification of subjects according to obesity and parameters analyzed revealed in the obesity group a median age of 10 years (8–12) and a BMI of 27.8 kg/m^2^ (24.85–30.4) (Table 1).

Paraclinical investigation of serum iron and ferritin levels showed a dramatic decrease in iron and an increase in ferritin in patients with obesity compared to the average weight group. There were no cases with high levels of serum iron or low levels of serum ferritin. Also, the other parameters analyzed to determine the presence of MetS had significantly altered values in the group with obesity (Table 1).

Analyzing the frequency distribution of cases, we observed the predominance of males in both average weight and obesity groups. The environment was predominantly rural for the average weight group and urban for the group with obesity. Regarding the imaging investigation, the degrees of hepatosteatosis identified showed an almost uniform distribution of cases in the group with obesity. The absence of hepatosteatosis in the average weight group, along with the presence of a single case in the obesity group, highlights the heterogeneity of the studied population (Table 2).

Deficit serum iron and increased serum ferritin were found in a large number of subjects diagnosed with obesity. MetS parameters were also modified in the group with obesity compared to the average weight group. The presence of MetS was identified in 64% of cases with obesity and in only 11.1% of patients in the other group (Table 2).

Borderline statistical correlations with obesity were found for parameters such as sex and environment. Parameters such as ultrasonography changes, serum iron deficiency, elevated serum ferritin, elevated abdominal perimeter (AP), elevated systolic blood pressure (SBP)/diastolic blood pressure (DBP), elevated triglycerides (TG), low high-density lipoprotein cholesterol (HDLch), and present MetS were statistically significantly correlated with obesity (Table 2).

### 2.2. MetS+ Group vs. MetS− Group

MetS was present in 34 patients, representing 50% of the study population. The distribution of cases according to the MetS criteria demonstrates that abdominal circumference, high triglyceride, and low HDLch are the three major criteria supporting the diagnosis. In our cohort, 27.9% of participants had no MetS criteria, while 22% met one or two criteria. Among those classified as MetS+, 23.5% met three criteria, 23.5% met four criteria, and 2.9% met all five criteria (Table 3).

**Figure 1:**
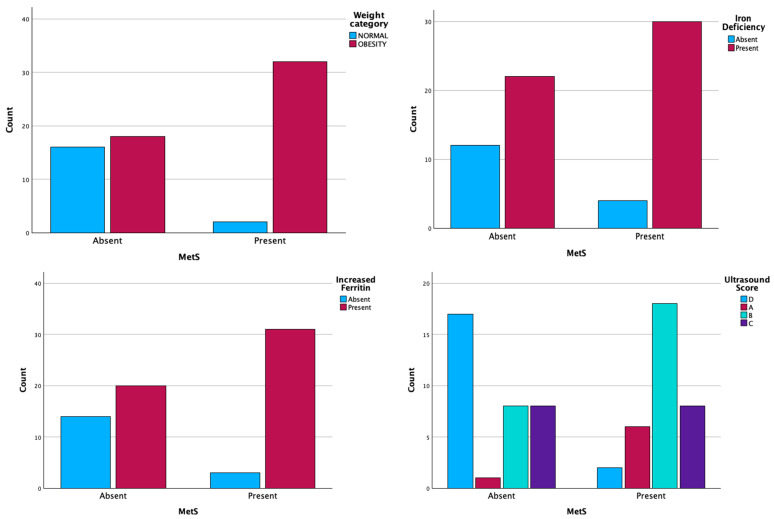
Distribution of patients according to the presence of MetS and statistical correlates weight, deficiency serum iron, increased serum ferritin, and ultrasound changes.

The present of MetS was statistically correlated with obesity, serum iron deficiency, increased serum ferritin levels, and ultrasonographic changes in the liver. The median age at diagnosis of MetS was 11 years and the BMI was 27.9 kg/m^2^ (Table 4, Figure 1).

A Spearman’s rank order correlation was conducted to examine the association between serum ferritin and serum iron and the presence of MetS. A moderate positive correlation was found between serum ferritin and the presence of MetS (ρ = 0.388, *p* = 0.001), suggesting that individuals with MetS tend to have higher ferritin levels. Serum iron exhibited a moderate negative correlation with MetS (ρ = −0.338, *p* = 0.005), showing that the presence of MetS is associated with lower serum iron levels.

A Jonckheere–Terpstra test was conducted to examine the trend in serum iron and serum ferritin levels across six ordered groups based on the number of MetS criteria (0 to 5) in order to determine if there is a trend in these parameters with the increase in MetS components. The results indicated a statistically significant trend for both serum iron (J-T = 550.000, *p* < 0.001) and serum ferritin (J-T = 1273.000, *p* < 0.001). The standardized J-T statistics (−3.902 for serum iron and 3.980 for serum ferritin) further confirm these significant associations. These findings suggest that as the number of MetS criteria increases, serum iron levels decrease, while, on the other side, serum ferritin levels show a progressive upward trend.

### 2.3. MetS and Ultrasonographic Changes

Out of the total, half of the patients with MetS were classified under Score B and 23.5% under Score C, indicating substantial hepatic alterations. Only 5.9% of MetS+ patients exhibited normal liver echogenicity (Score D), while among MetS- patients, 50% had normal liver structure (Score D). Also, only 23.5% were classified as Score C, and 23.5% as Score B, suggesting a lower prevalence of steatosis (Table 4).

**Figure 2:**
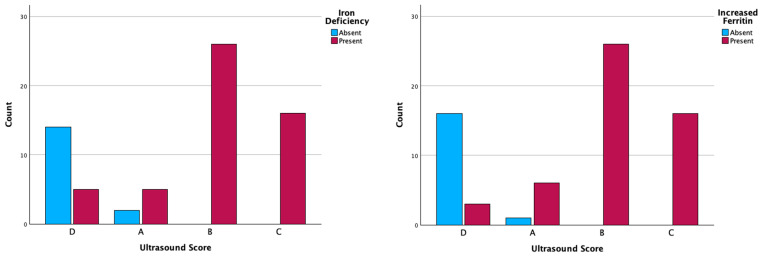
The distribution of cases according to iron deficiency and increased ferritin and imaging evaluation.

Of all patients included in this study, 16 (23.5%) cases had normal serum iron level, 2 (2.9%) cases were found with ultrasonographic Score A, and 14 (20.5%) with Score D. Deficiency serum iron was predominantly observed in patients with ultrasonographical changes, an aspect that is statistically correlated (Table 5, Figure 2).

Of the total, 17 (25%) patients with normal serum ferritin levels had Score A 1 (1.4%) and Score D 16 (23.5%), respectively. Increased serum ferritin was statistically correlated with ultrasonographic changes (Table 5, Figure 2).

A Jonckheere–Terpstra test was conducted to examine the trend in scores across ultrasound changes among MetS number of components in order to determine if there is a statistically significant trend in performance across these groups. These results imply that the ordered groups (0 MetS components to 5 MetS components) exhibit a statistically significant trend in scores (J-T = 3.367, *p* < 0.001). These findings suggest that increasing number of MetS criteria determines a higher ultrasound score and, respectively, more fat accumulation in the liver.

Also, the same test was used to assess the presence of a monotonic trend in serum iron and serum ferritin levels across the ultrasound score categories representing the severity of hepatic steatosis. The results indicated a significant negative trend in serum iron levels as the ultrasound score increased (J-T = 122.000, standardized J-T = −7.796, *p* < 0.001), suggesting that higher hepatic fat accumulation is associated with lower serum iron concentrations. On the other side, serum ferritin levels exhibited a significant positive trend across ultrasound categories (J-T = 1465.500, standardized J-T = 7.184, *p* < 0.001), demonstrating that ferritin levels progressively increase with the severity of hepatic steatosis.

## 3. Discussion

Abnormal weight gain, generally known under the terms overweight and obesity, represents abnormal or excessive fat accumulation, presenting a long-term health risk [2]. BMI, calculated from height and weight, classifies individuals into weight categories and helps assess the risk for chronic diseases. Childhood BMI also predicts future health outcomes, while additional measurements such as abdominal circumference and waist-to-hip ratio complement BMI in evaluating risks for persistent conditions [26]. Even if it is not a direct measure of body fat, it does not displace clinical judgment [27]. In the pediatric population, due to the continuous changes in a child’s body weight and height, BMI is interpreted based on age- and sex-specific percentiles, which help clinicians classify individuals into categories such as underweight, healthy weight, overweight, obesity, and severe obesity, accounting for natural growth and developmental differences [28,29].

In our study, we categorized subjects into two main groups: those with and without obesity and those with and without MetS. As expected, the BMI was significantly higher in the obesity group compared to the average weight group, in line with its definition. Furthermore, when comparing subjects with and without MetS, the BMI was notably higher in those with MetS+. This highlights that excessive weight gain, particularly the accumulation of adipose tissue, predisposes individuals to metabolic dysregulation and may act as a key trigger for its development.

According to some authors, childhood obesity is the main driver for impaired insulin sensitivity, strongly linked to MetS, with evidence showing weight gain exacerbating insulin resistance and weight loss improving it [7]. However, other authors argue that while obesity is undoubtedly a major factor in MetS progression, it is not the primary cause, as the metabolic dysregulations characteristic of MetS can also be observed in individuals with healthy weight [30]. The more incriminating factor for these changes is poor diet (high in ultra-processed foods, added sugars, and unhealthy fats), influencing metabolic health beyond the effects of obesity alone. Poor dietary patterns contribute to insulin resistance, oxidative stress, and systemic inflammation, key drivers of metabolic dysfunction [31].

In our study, obesity was significantly correlated with MetS, with a high prevalence of this condition among subjects with obesity (64%) compared to those with average weight (11.1%). A recent meta-analysis by Wentzel et al. supports the fact that children and adolescents with excessive weight have a greater predisposition to developing MetS [32]. Another finding in our study is that abdominal circumference, triglycerides, and HDLch were the most relevant diagnostic criteria for MetS, which is also supported by other similar studies [33].

Numerous studies in both pediatric and adult populations have confirmed the inflammatory nature of MetS and obesity, highlighting their contribution to metabolic dysfunction. Elevated circulating biomarkers further link these conditions to long-term complications such as type 2 diabetes and atherosclerotic cardiovascular disease [34]. Leukocyte counts, C-reactive protein (CRP), interleukin-6 (IL-6), interleukin-10, and tumor necrosis factor-α (TNF-α) are the markers used in most studies that are determined to be associated with low-grade chronic inflammation [17,35,36,37,38].

One key role in the healthy development of a child is iron, which is dependent on the intake and its absorption in the duodenum [39]. Hepcidin is the main regulator of iron metabolism, and it is upregulated by proinflammatory cytokines such as IL-6, which have shown to be high in conditions like obesity and MetS, leading to reduced iron absorption and availability, which leads to iron deficiency in these individuals [40,41,42]. In our study, we observed a significant alteration in iron metabolism, with a decrease in serum iron levels among the groups with obesity/overweight, MetS+, and alterations in the ultrasonographic score that determines the severity of hepatic steatosis, which indirectly proves that hepcidin overproduction, induced by the low-grade chronic inflammation present in these conditions, limits iron absorption. However, weight loss interventions have been shown to restore iron homeostasis, suggesting that serum hepcidin quantification could be a valuable tool in assessing iron deficiency in this population [43].

Ferritin is a key protein in iron homeostasis, serving as an intracellular iron storage molecule. It also functions as an acute-phase reactant, with synthesis upregulated by pro-inflammatory cytokines, particularly IL-6, independently of iron status [44]. Activation of the IL-6–hepcidin axis during chronic low-grade inflammation promotes increased ferritin levels and reduced intestinal iron absorption, contributing to functional iron deficiency despite sufficient or elevated systemic iron stores [43,44]. Consequently, ferritin plays an essential role as a marker of oxidative stress, being involved in regulating iron metabolism and preventing its toxicity by storing it in an inert form. However, excess intracellular iron stimulates the Fenton reaction and the formation of reactive oxygen species, which is associated with metabolic disorders, affecting mitochondrial functions, lipid metabolism, and insulin signaling, which can lead to cell damage and death [45].

Supporting our findings, previous studies have reported a positive association between increased serum ferritin levels and, with ferritin correlating with insulin resistance, dyslipidemia (elevated cholesterol, low-density lipoprotein cholesterol (LDLch), triglycerides, and low HDLch), and individual MetS components. These results further suggest that altered iron metabolism may play a role in metabolic dysfunction [46]. We determined that ferritin significantly increases with the number MetS components, which translates to an increase on inflammation with MetS severity.

**Figure 3:**
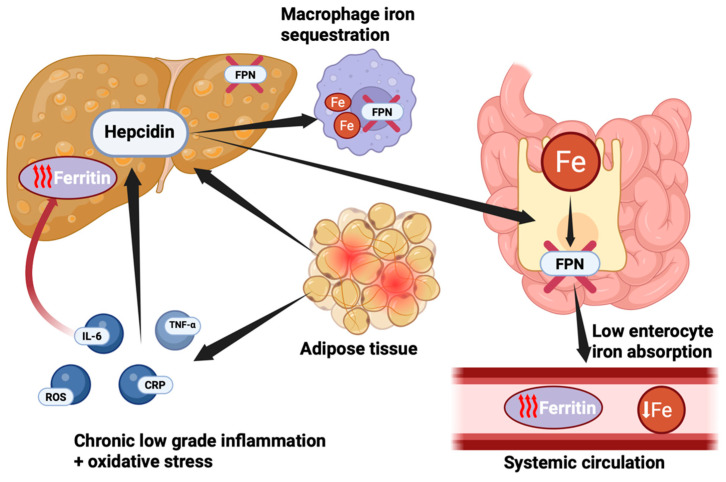
Pathophysiological mechanism linking adipose tissue, chronic low-grade inflammation, and disorders in iron metabolism observed in children with obesity and MetS. Excess adipose tissue contributes to a pro-inflammatory state, characterized by elevated production of cytokines such as IL-6 and tumor necrosis factor-alpha (TNF-α), along with increased oxidative stress markers (reactive oxygen species—ROS) and C-reactive protein (CRP). These inflammatory mediators stimulate hepatic synthesis of hepcidin, a key regulator of systemic iron. It binds to ferroportin (FPN)—a cellular iron exporter expressed by enterocytes and hepatic macrophages—thereby reducing intestinal iron absorption and promoting iron sequestration in macrophages. As a consequence, we encounter low serum iron. In response to this functional iron deficiency and persistent inflammatory state, ferritin synthesis is upregulated as part of the acute-phase response. This explains the paradoxical finding of elevated serum ferritin despite low circulating iron levels in children with obesity and MetS. Created with https://www.biorender.com (accessed on 12 April 2025).

To further clarify the mechanisms underlying these findings, we provide a schematic illustration of the pathophysiological sequencing linking adiposity, inflammation, and iron dysregulation (Figure 3).

Our exclusion criteria eliminated patients with autoimmune, infectious, or chronic inflammatory diseases, which minimized potential confounders that could independently elevate ferritin levels or influence iron metabolism. Therefore, the observed combination of elevated ferritin and reduced serum iron levels in our MetS + and obesity groups is mainly driven by obesity-associated low-grade inflammation and the consequent activation of the IL-6–hepcidin axis, rather than by other inflammatory conditions.

Nowadays, NAFLD, which translates as fat accumulation in the liver, is the most common chronic liver disease in children, strongly linked to obesity, with recent meta-analyses estimating a 6.3% prevalence in the general pediatric population and over 40% in children with overweight/obesity. Given its metabolic associations, an international panel proposed renaming NAFLD to MASLD, emphasizing its link to metabolic dysfunction [47,48]. The easiest, most cost-effective, and non-invasive way to estimate hepatic fat infiltration in any patient is through liver ultrasound. While it is not a perfect method due to variations in sensitivity, specificity, and subjectivity being an operator-dependent method, it remains the preferred technique for both adults and children, as it is excellent in disease progression and identifying subjects at risk for complications [49].

Typically, liver echogenicity is similar to renal cortex or spleen parenchymal echogenicity, and the intrahepatic structures are well delimited. In conditions such as obesity and MetS, fat accumulates in the liver, leading to increased echogenicity. As fat deposition progresses, vessels and bile ducts become less distinguishable, and the diaphragm appears blurred due to depth-dependent signal reduction [50].

Liver ultrasound findings in our study demonstrated a significant association between MetS and hepatic alterations, reinforcing the established link between MetS and hepatic steatosis. Liver fat deposition was quantified using an ultrasound scoring system, where increasing scores (A–C) reflected greater hepatic steatosis, and Score D indicated normal liver echogenicity. Among patients with MetS, the majority exhibited hepatic steatosis, with 36.7% classified as Score B, 23.5% as Score C, and 10.2% as Score A, while only 4.4% retained normal liver echogenicity (Score D). Our study focused on steatosis; however, as evidence shows, progressive hepatic steatosis may precede fibrosis. Therefore, non-invasive fibrosis assessment using the pediatric NAFLD fibrosis score or elastography [51,52] could provide additional prognostic information and should be considered in future research.

Furthermore, serum iron deficiency was significantly correlated with more advanced ultrasonographic liver changes, with 36.7% of cases classified as Score B and 23.5% as Score C. These findings align with previous studies indicating that hepatic steatosis is a frequent comorbidity in children with obesity and MetS, often reflecting the severity of metabolic dysfunction [49,53]. The strong correlation between increased serum ferritin, iron deficiency, and hepatic steatosis severity highlights the importance of iron homeostasis in the progression of these conditions in pediatric patients.

Our study has several limitations that should be considered when interpreting the results. First, the small sample size, the limited ethnic diversity in participants (non-Hispanic white/Caucasian), and the fact that it was conducted in a single center limits the generalizability of the findings to a broader pediatric population. Additionally, the retrospective nature of this study and the lack of follow-up prevent a comprehensive assessment of the progression and impact of inflammation. Another limitation is the absence of advanced diagnostic techniques, such as liver biopsy or MRI (magnetic resonance imaging), which could have provided more precise confirmation of hepatic alterations. However, we aimed to use ultrasound, as it is non-invasive, reproductible, low-cost, and is broadly accessible.

Therefore, future studies should focus on larger, more diverse cohorts to improve the generalizability of results and to confirm the observed metabolic and inflammatory alterations. Prospective, longitudinal studies are a better study design in order to clarify the temporal progression and causal links from obesity to MetS, subsequent iron dysregulation, and hepatic complications, which could further help to implement more targeted preventive and therapeutic strategies.

By addressing these gaps, future studies can contribute to a deeper understanding of obesity-related metabolic dysfunctions and support the development of targeted strategies to improve pediatric health outcomes.

Finally, we recommend that ferritin and iron serum levels, as good markers of iron metabolism and inflammation, to be included in the assessment of children and adolescents with obesity and MetS. Their low cost, wide availability, and ease of interpretation makes them valuable tools in the management and monitorization in these conditions.

## 4. Materials and Methods

### 4.1. Study Design

This is a retrospective study designed to investigate the relations between MetS, serum iron, and ferritin status in children and adolescents. We also investigated hepatic structural changes identified by ultrasound. The patients included in this study were evaluated based on epidemiological, clinical, and paraclinical data, which were then statistically compared.

### 4.2. Study Population

We included 68 children aged between 6 and 14 years, 50 subjects with obesity and 18 with average weight. Patients were selected and evaluated between January 2023 and September 2024 in the pediatric department of Craiova County Emergency Clinical Hospital.

### 4.3. Inclusion Criteria

Inclusion criteria for the study group were children and adolescents age between 6 and 14 years. Obesity was defined according to World Health Organization (WHO) criteria for age and gender, with BMI ≥ 95th percentile classified as obesity, and average weight defined as BMI between the 5th and 85th percentile. For our study, those with a BMI between the 85th and 95th percentile, usually classified as overweight, were analyzed alongside with the obesity group.

### 4.4. Exclusion Criteria

All individuals with inflammatory, autoimmune, or other liver diseases like viral hepatitis (B virus infection with or without D virus, C virus infection), as well as those with endocrine or genetic disorders or other chronic conditions, were excluded from this study. Additionally, patients with congenital malformations, recent infectious pathology (within the last three months), immunocompromised status, or those receiving antibiotic or iron supplement treatment in the past three months were also excluded.

We also excluded any patients who did not have all the data required for our study such as those missing laboratory tests, with incomplete measurements, or not having had a liver ultrasound examination.

### 4.5. Patient Assessment

Identification of non-eligible cases was based on the history of the patients. Epidemiological data referring to age, gender, and environment were recorded. Investigation of the patients included in this study was based on the determination of obesity, respectively, BMI (height, weight, age, sex), investigation of MetS criteria, liver ultrasound evaluation, and serum iron and ferritin levels. The criteria for diagnosing MetS, recognized as a complex clinical entity, included measurements of AP, blood pressure (BP), TG, HDLch, and blood glucose levels.

### 4.6. Measurements

Anthropometric measurements were performed using standardized methods. Measurements were taken in the morning before any food or liquid ingestion. For height, a thaliometer with 1 mm graduation was used, and for weight measurement we used an electronic scale (100 g graduation). BMI was calculated by dividing body weight (expressed in kilograms) by height squared (expressed in meters), taking into account gender and age. The waist circumference was measured at the end of the expiration in the umbilical area with a thaliometer for circumferences (graduation 1 mm), after which this was transformed into percentiles according to recommendations for evaluation of overweight/obese children, the percentiles above 90 being a risk factor for MetS in the case of obese patients [54].

Blood pressure was measured three times after a rest period of ten minutes with an electronic tensiometer, and the average value was obtained. Blood pressure percentiles were calculated according to the United States National High Blood Pressure Education Program Working Group guidelines, using the online calculators provided by the MSD Manual Professional Version, according to age, gender, and height [55,56]. For both systolic and diastolic blood pressure values, the percentile below 90% meant normal pressure; between 90% and 95%, prehypertension; between 95% and 99%, grade I hypertension; and over 99%, grade II hypertension [57]. For easy classification and interpretation of the data, all the subjects with altered blood pressure were classified as having hypertension.

### 4.7. Laboratory Tests

A venous blood sample was collected from each participant by phlebotomy after a fasting period of at least 8 h (between 8 and 12 h) by an experienced nurse and TG, HDLch, blood glycemia, and serum iron and serum ferritin were analyzed.

Iron deficiency was defined as serum iron levels below the reference values as follows:

- Children < 14 years: 29–137 μg/dL;
- Boys (14–19 years): 43–176 μg/dL;
- Girls (14–19 years): 33–170 μg/dL.

Serum ferritin reference values were as follows:

- 4–7 years: 4–67 ng/mL;
- 7–13 years:
  ○ Males: 14–124 ng/mL;
  ○ Females: 7–84 ng/mL;
- 13–18 years:
  ○ Males: 14–152 ng/mL;
  ○ Females: 13–68 ng/mL.

Both serum iron and ferritin serve as indirect markers for evaluating inflammation associated with obesity.

### 4.8. Definition of MetS

The presence of MetS was defined by the fulfilment of at least three of the following criteria:

- Abdominal obesity—increased AP (≥90th percentile of waist circumference for patients of the same age and gender).
- Hypertriglyceridemia—TG ≥ 100 mg/dL for children aged 0–9 years and ≥130 mg/dL for those aged 10–19 years.
- Low HDL cholesterol—HDLch < 40 mg/dL.
- Hypertension—SBP or DBP ≥ 90th percentile for children of the same age, height, and gender.
- Fasting hyperglycemia—glycemia ≥ 100 mg/dL [58,59,60,61,62].

Next, we split the subjects in groups based on MetS diagnosis, categorizing individuals as MetS+ (those meeting ≥ 3 criteria as for MetS definition) and MetS− (those meeting ≤ 2 criteria as for MetS definition), as well as according to the number of MetS criteria met (ranging from 0 to 5), in order to fulfil our objectives.

### 4.9. Imagistic Result

Imaging data were obtained using ultrasound performed with an age-adapted scanning transducer by an experienced pediatrician. In order to quantify liver fat deposition, we classified patients as follows:

- Score A: diffuse increased liver echogenicity; periportal and diaphragmatic echogenicity is still appreciable;
- Score B: diffuse increased liver echogenicity hiding periportal echogenicity but diaphragmatic echogenicity is still appreciable;
- Score C: diffuse increased liver echogenicity hiding periportal and diaphragmatic echogenicity;
- Score D: normal liver echogenicity [63].

This ultrasound scoring system has been validated in other pediatric populations and is recognized for its reproducibility, although operator experience remains an important factor influencing accuracy [49,63].

### 4.10. Statistical Analysis

The clinicoepidemiological data were analyzed in an integrated manner using the chi-square test (χ^2^) in IBM SPSS Statistics for Macintosh, Version 30 (Statistical Package for the Social Sciences), with a significance threshold of *p* < 0.05. Additionally, this study evaluated trends of association between various parameters, even in cases where the relationships did not reach statistical significance.

The Shapiro–Wilk test of normality was conducted to determine whether age, BMI, AP, serum ferritin, SBP, DBP, glycemia, TG, HDLch, and serum iron were normally distributed. The results indicate that we must reject the null hypothesis for all variables except for glycemia (*p* = 0.115) and AP, which are the only ones with a normal distribution. Hence, nonparametric statistical tests were chosen for further analysis to ensure the validity of the results. In line with this approach, data for variables without a normal distribution were presented as median values with interquartile range (25–75th percentile) to accurately reflect their distribution.

## 5. Conclusions

Our findings confirm the association of pediatric obesity, hepatic steatosis, and MetS. Abdominal circumference, triglycerides, and HDLch were the most relevant indicators for MetS. Additionally, we observed significant alterations in iron metabolism, with lower serum iron levels and increased ferritin levels correlating with the severity of MetS and hepatic steatosis.

Liver ultrasound findings reinforced the link between MetS and hepatic alterations, demonstrating a progressive increase in hepatic echogenicity with worsening metabolic status.

The relationship between iron metabolism markers and hepatic steatosis severity further supports the role of ferritin as an accessible biomarker for identifying children at risk of metabolic and hepatic complications. These insights highlight the potential utility of ferritin in clinical screening and risk stratification.

Future prospective, longitudinal studies are necessary to clarify the causal pathways from obesity to metabolic dysfunction and hepatic alterations, and to inform targeted prevention and intervention strategies in pediatric populations. 

## Figures and Tables

**Table 1:** The distribution of cases in relation to the analyzed parameters.

Parameters	Average Weight (n = 18)	Obesity (n = 50)
Age (years)	11 (8.5–12)	10 (8–12)
BMI (kg/m^2^)	17.3 (16.57–18.25)	27.8 (24.85–30.4)
Serum iron (μg/dL)	73 (41.25–83.25)	10 (7–12)
Serum ferritin (ng/mL)	41 (31.5–55)	203 (185.5–221)
Height (cm)	138 (126.5–146.25)	144.5 (134–156.25)
AP (cm)	73.2 ± 6.3	90 ± 12.1
SBP (mmHg)	108 (105–112.5)	119 (109.75–130.25)
DBP (mmHg)	59 (57.75–61.25)	74 (64–83.5)
Glycemia (mg/dL)	70.2 ± 8.1	87.6 ± 9.4
HDLch (mg/dL)	59.37 (57.1–63.87)	29.16 (22.91–42.65)
TG (mg/dL)	108 (88–115.25)	161.5 (99–223.75)

Data are presented as mean ± standard deviation for normally distributed variables and as median (25–75th percentile) for non-normally distributed variables. BMI: body mass index; AP: abdominal perimeter; SBP: systolic blood pressure; DBP: diastolic blood pressure; HDLch: high-density lipoprotein cholesterol; TG: triglycerides.

**Table 2:** The distribution of cases in relation to weight and the analyzed parameters.

Parameters (No. Cases) (%)		Average Weight (n = 18)	Obesity (n = 50)	*p*-Value (x^2^ Test)
Gender	Female	2 (11.1%)	16 (32%)	*p* = 0.08
	Male	16 (88.9%)	34 (68%)	
Environment	Urban	6 (33.3%)	31 (62%)	*p* = 0.03
	Rural	12 (66.7%)	19 (38%)	
Ultrasonography	Score A	0	8 (16%)	*p* < 0.001
	Score B	0	25 (50%)	
	Score C	0	16 (32%)	
	Score D	18 (100%)	1 (2%)	
Deficit serum iron		4 (22.2%)	48 (96%)	*p* < 0.001
Increased serum ferritin		2 (11.1%)	49 (98%)	*p* < 0.001
High AP		2 (11.1%)	46 (92%)	*p* < 0.001
High SBP	p90–95	0	3 (6%)	*p* = 0.01
	p95–99	0	7 (14%)	
	p > 99	0	11 (22%)	
High DBP	p90–95	0	4 (8%)	*p* = 0.01
	p95–99	0	10 (20%)	
	p > 99	0	7 (14%)	
High SBP/DBP		0	27 (54%)	*p* < 0.001
High Glycemia		0	4 (8%)	*p* > 0.05
High TG		2 (11.1%)	29 (58%)	*p* = 0.001
Low HDLch		2 (11.1%)	35 (70%)	*p* < 0.001
MetS +		2 (11.1%)	32 (64%)	*p* < 0.001

AP: abdominal perimeter/circumference; SBP: systolic blood pressure; DBP: diastolic blood pressure; TG: triglycerides; HDLch: high-density lipoprotein cholesterol; MetS: metabolic syndrome; MetS +: present metabolic syndrome.

**Table 3:** The distribution of subjects and their parameters in relation to the presence of MetS.

Parameters (No. Cases) (%)		MetS + (n = 34)	MetS − (n = 34)
AP	High	34 (100%)	14 (41.2%)
	cm	92.8 ± 11.3	78.2 ± 10.7
High SBP	p90–95	3 (8.8%)	0 (0%)
	p95–99	5 (14.7%)	2 (5.9%)
	p > 99	9 (26.5%)	2 (5.9%)
SBP	mmHg	120 (111.75–132.25)	109 (105.75–113.25)
High DBP	p90–95	3 (8.8%)	1 (2.9%)
	p95–99	8 (23.5%)	2 (5.9%)
	p > 99	7 (20.6%)	0 (0%)
DBP	mmHg	79 (66.75–95.25)	62 (58–66)
SBP/DBP	High	22 (64.7%)	5 (14.7%)
Glycemia	High	3 (8.8%)	1 (2.9%)
	mg/dL	87.7 ± 9.6	78.2 ± 12.2
TG	High	31 (91.2%)	0 (50%)
	mg/dL	204.5 (157.75–247.5)	99 (92.75–109)
HDLch	Low	32 (94.1%)	5 (14.7%)
	mg/dL	27.29 (22.62–34.03)	56.28 (43.17–61.82)
MetS number of criteria	0	-	19 (27.9%)
	1	-	6 (8.8%)
	2	-	9 (13.2%)
	3	16 (23.5%)	-
	4	16 (23.5%)	-
	5	2 (2.9%)	-

Data are presented as mean ± standard deviation for normally distributed variables and as median (25–75th percentile) for non-normally distributed variables. AP: abdominal perimeter/circumference; SBP: systolic blood pressure; DBP: diastolic blood pressure; TG: triglycerides; HDLch: high-density lipoprotein cholesterol; MetS: metabolic syndrome; MetS +: present metabolic syndrome; MetS −: absent metabolic syndrome.

**Table 4:** The distribution of cases in relation to present/absent MetS and the analyzed parameters.

Parameters (No. Cases) (%)		MetS + (n = 34)	MetS − (n = 34)	*p*-Value
Age (years)		11 (9–12)	9 (7–12)	-
BMI (kg/m^2^)		27.9 (24.85–30.8)	20.8 (17.52–26.35)	*p* < 0.001
Average weight		2 (5.9%)	16 (47.1%)	
Obesity		32 (94.1%)	18 (52.9%)	
Serum iron	μg/dL	10 (7–12.5)	17 (9–67)	*p* = 0.02
	Deficit	30 (88.2%)	22 (64.7%)	
Serum ferritin	ng/mL	203.5 (176.75–223)	166 (41.75–203.25)	*p* = 0.002
	Increased	31 (91.2%)	20 (58.8%)	
Ultrasound	Score A	7 (20.6%)	1 (2.9%)	*p* < 0.001
	Score B	17 (50%)	8 (23.5)	
	Score C	8 (23.5)	8 (23.5)	
	Score D	2 (5.9%)	17 (50%)	

Data are presented as mean ± standard deviation for normally distributed variables and as median (25–75th percentile) for non-normally distributed variables. BMI: body mass index; MetS: metabolic syndrome; MetS: metabolic syndrome; MetS +: present metabolic syndrome; MetS −: absent metabolic syndrome.

**Table 5:** The distribution of cases in relation to the ultrasonographic score assessment and iron and ferritin levels.

Parameters (No. Cases) (%)			Ultrasonographic Score			*p*-Value
		A (n = 8)	B (n = 25)	C (n = 16)	D (n = 19)	
Serum iron	Normal level	2 (2.9%)	0	0	14 (20.5%)	*p* < 0.001
	Deficit	6 (8.8%)	25 (36.7%)	16 (23.5%)	5 (7.3%)	
Serum ferritin	Normal level	1 (1.4%)	0	0	16 (23.5%)	*p* < 0.001
	Increased	7 (10.2%)	25 (36.7%)	16 (23.5%)	3 (4.4%)	

## Data Availability

The authors decided not to share the data.

## Abbreviations

The following abbreviations are used in this manuscript:

MetS	metabolic syndrome
BMI	body mass index
HDLch	high-density lipoprotein cholesterol
SBP	systolic blood pressure
DBP	diastolic blood pressure
TG	triglycerides
AP	abdominal perimeter
CDC	Centers for Disease Control and Prevention
WHO	World Health Organization
LDLch	Low-density lipoprotein cholesterol
NAFLD	nonalcoholic fatty liver disease
MAFLD	metabolic-associated fatty liver disease
MRI	magnetic resonance imaging
IL-6	interleukin-6
CRP	C-reactive protein
TNF-α	tumor necrosis factor-alpha
FPN	ferroportin
ROS	reactive oxygen species
Fe	iron
